# Mobile clinics in humanitarian emergencies: a systematic review

**DOI:** 10.1186/s13031-020-0251-8

**Published:** 2020-01-30

**Authors:** Catherine R. McGowan, Louisa Baxter, Claudio Deola, Megan Gayford, Cicely Marston, Rachael Cummings, Francesco Checchi

**Affiliations:** 1 Humanitarian Public Health Technical Unit, Save the Children UK, London, UK; 2 Faculty of Public Health & Policy, London School of Hygiene & Tropical Medicine, 15-17 Tavistock Place, WC1H 9SH, London, UK; 3 Faculty of Epidemiology & Population Health, London School of Hygiene & Tropical Medicine, London, UK

**Keywords:** Mobile clinics, Mobile health clinics, Mobile health units, Humanitarian, Systematic review

## Abstract

**Background:**

Despite the widespread reliance on mobile clinics for delivering health services in humanitarian emergencies there is little empirical evidence to support their use. We report a narrative systematic review of the empirical evidence evaluating the use of mobile clinics in humanitarian settings.

**Methods:**

We searched MEDLINE, EMBASE, Global Health, Health Management Information Consortium, and The Cochrane Library for manuscripts published between 2000 and 2019. We also conducted a grey literature search via Global Health, Open Grey, and the WHO publication database. Empirical studies were included if they reported on at least one of the following evaluation criteria: relevance/appropriateness, connectedness, coherence, coverage, efficiency, effectiveness, and impact.

**Findings:**

Five studies met the inclusion criteria: all supported the use of mobile clinics in the particular setting under study. Three studies included controls. Two studies were assessed as good quality. The studies reported on mobile clinics providing non-communicable disease interventions, mental health services, sexual and reproductive health services, and multiple primary health care services in Afghanistan, the Democratic Republic of the Congo , Haiti, and the Occupied Palestinian Territories. Studies assessed one or more of the following evaluation domains: relevance/appropriateness, coverage, efficiency, and effectiveness. Four studies made recommendations including: i) ensure that mobile clinics are designed to complement clinic-based services; ii) improve technological tools to support patient follow-up, improve record-keeping, communication, and coordination; iii) avoid labelling services in a way that might stigmatise attendees; iv) strengthen referral to psychosocial and mental health services; v) partner with local providers to leverage resources; and vi) ensure strong coordination to optimise the continuum of care. Recommendations regarding the *evaluation* of mobile clinics include carrying out comparative studies of various modalities (including fixed facilities and community health workers) in order to isolate the effects of the mobile clinics. In the absence of a sound evidence base informing the use of mobile clinics in humanitarian crises, we encourage the integration of: i) WASH services, ii) nutrition services, iii) epidemic surveillance, and iv) systems to ensure the quality and safety of patient care. We recommend that future evaluations report against an established evaluation framework.

**Conclusion:**

Evidence supporting the use of mobile clinics in humanitarian emergencies is limited. We encourage more studies of the use of mobile clinics in emergency settings.

**Funding:**

Salary support for this review was provided under the RECAP project by United Kingdom Research and Innovation as part of the Global Challenges Research Fund, grant number ES/P010873/1.

## Background

Humanitarian emergencies are typically characterised by excess morbidity and mortality due to various emergent risk factors including: population displacement, widespread damage to societies and economies, and the need for large-scale humanitarian assistance. Providing essential health services during a humanitarian emergency is complicated by disrupted health systems, damaged infrastructure, and reduced care-seeking behaviors resulting from eroded social support mechanisms [1, 2]. Humanitarian emergencies present unique challenges for health service delivery including, but not limited to, sudden changes in the nature and extent of the health burden (potentially requiring triage and urgent referral), restricted access to services, and a heightened need to implement safeguarding measures.

The Sphere minimum standards for health service delivery require that, “people have access to integrated quality healthcare that is safe, effective and patient-centered” ([1], p., 298), with a suggested target of ≥80% of the population able to access primary healthcare within a one hour walk [1]. Healthcare can be delivered using different strategies including community-level interventions (e.g. community health workers), fixed healthcare facilities, and mobile clinics. Mobile clinics (a.k.a. mobile health clinics, mobile health units) are intermittent ambulatory health services which typically include a combination of preventive (e.g. vaccination, screening, and health promotion) and curative services. Mobile clinics are a common modality for delivering health services in humanitarian  emergencies. Despite this, there is a paucity of robust empirical evidence to support the design and implementation of mobile clinics in humanitarian settings.

The main objective of mobile clinics is to improve access to healthcare [3, 4] by providing a package of limited primary health services, with referral to nearby fixed structures for conditions not manageable under this package [3]. The term ‘mobile clinic’ is often used to describe both mobile health services as well as mobile outreach services (i.e. services which advance a health service from an existing health centre); though mobile outreach services are perhaps best considered a distinct modality. Mobile clinics may function in tandem with, and in support of, community health care workers in order to further extend access to services. It is recommended that mobile clinics remain an exceptional modality, only employed as a “last resort” to reach populations cut off from health services [3]. Mobile clinics are expensive (relative to other delivery strategies), logistically burdensome, time-inefficient (a large portion of productive time is spent travelling), and rarely demonstrate a lasting impact [3–5]. Due to issues relating to sustainability they are often ill-suited to addressing chronic diseases. Depending on the frequency with which clinics visit communities, they may also offer limited coverage for addressing acute illnesses. The World Health Organization considers mobile clinics “a good illustration of the tension between equity of access and the efficient utilization of scarce human resources”. ([5], p., 14) Despite these limitations, mobile clinics are endorsed for use in humanitarian crises by agencies and donors who are eager to support their implementation [6].

In response to increasing pressure from agencies and donors to implement and support mobile clinics in humanitarian responses, we sought to review the literature evaluating the mobile clinic modality in humanitarian settings. We also aimed to review recommendations for improving the design and evaluation of mobile clinics in humanitarian settings. Finally, we address clear gaps in the design of mobile clinics in the absence of a robust body of empirical evidence to inform their use.

The review protocol has been published in the PROSPERO prospective review registry (#132888) [7]. This review is reported against the Preferred Reporting Items for Systematic Reviews and Meta-Analyses (PRISMA) checklist [8].

## Main text

### Methods

We carried out a narrative systematic review of the empirical evidence for the use of mobile clinics in humanitarian crises.

#### Search strategy

The search was carried out on 3 April 2019 via Ovid (MEDLINE, EMBASE, Global Health, and The Health Management Information Consortium) and the Cochrane Library. We used a combination of MeSH/EMTREE terms (for MEDLINE and EMBASE) and keywords to identify all literature published in any language between 1 January 2000 and 3 April 2019. We searched ‘all fields’ for: “mobile clinic*”, “mobile health clinic*”, and “mobile health unit*”; and searched for the MeSH and EMTREE Heading “Mobile Health Units” (MeSH UID D008952). We also searched ClinicalTrials.gov and the WHO International Clinical Trials Registry Platform using the search string: (mobile clinics OR mobile units OR mobile health units OR mobile health clinics) AND (humanitarian OR emergency). In addition to Global Health (which indexes grey literature) we searched Open Grey (http://www.opengrey.eu/), and the World Health Organization publications database (https://apps.who.int/iris) for grey literature using only the mobile clinic search terms above (i.e. not ‘humanitarian’ or ‘emergency’). We also carried out an internet search using the DuckDuckGo search engine (https://duckduckgo.com/).

CRM designed and executed the searches. CRM and LB screened the retrieved sources; FC screened sources where there was lack of consensus.

#### Eligibility criteria

Sources were included if they reported on an empirical study (qualitative or quantitative) of the effectiveness of mobile clinics (defined broadly to include mobile outreach services) in the context of a humanitarian emergency. Eligible studies contained data relating to clinic attendees or clinic staff.

Studies were excluded if they reported on fixed or semi-fixed facilities (including field hospitals), mobile clinics providing laboratory or diagnostic services only, or those providing dental or ophthalmology services. We did not include mobile clinics that operate exclusively via air (e.g. air ambulance) or water transport (e.g. medical ships) even if they offer similar services to land units, as these are not the modalities currently prioritised by agencies and donors. We excluded studies reporting on the effectiveness of mobile clinics outside humanitarian responses. We did not include papers reporting solely on the number/type of services provided, or the demographics of beneficiaries and/or the catchment population.

#### Data extraction and quality assessment

We used the widely adopted Organization for Economic Cooperation-Development Assistance Committee evaluation criteria (OECD-DAC) - adapted for humanitarian contexts by the Active Learning Network for Accountability and Performance in Humanitarian Action (ALNAP) - to define the following evaluation domains: relevance/appropriateness, connectedness, coherence, coverage, efficiency, effectiveness, and impact [9].

*Relevance/appropriateness*: “…relevance is concerned with assessing whether the project is in line with local needs and priorities (as well as donor policy) [and] appropriateness is the tailoring of humanitarian activities to local needs…”. ([9], p., 20) In addition, others have suggested expanding the definition to include other considerations including the needs of vulnerable groups, relevance in the face of evolving needs, and appropriateness to crisis contexts [10, 11]. *Connectedness* refers to, “…the need to ensure that activities of a short-term emergency nature are carried out in a context that takes longer-term and interconnected problems into account”. ([9], p., 20) *Coherence* is defined as, “[t]he need to assess security, developmental, trade and military policies as well as humanitarian policies, to ensure that there is consistency and, in particular, that all policies take into account humanitarian and human-rights considerations. ([9], p., 21) *Coverage* is defined as, “[t]he need to reach major population groups facing life-threatening suffering wherever they are”. ([9], p., 21) *Efficiency* measures, “…the outputs – qualitative and quantitative – achieved as a result of inputs. This generally requires comparing alternative approaches to achieving an output, to see whether the most efficient approach has been used”. ([9], p., 21) *Effectiveness* measures, “… the extent to which an activity achieves its purpose, or whether this can be expected to happen on the basis of the outputs [;] implicit within the criterion of effectiveness is timeliness”. ([9], p., 21) Finally, *impact* considers, “…the wider effects of the project – social, economic, technical, environmental – on individuals, gender- and age-groups, communities and institutions. Impacts can be intended and unintended, positive and negative, macro (sector) and micro (household)”. ([9], p., 21)

CRM extracted data into a framework including both descriptive domains (e.g. setting, outcomes, recommendations) and the OECD-DAC evaluation criteria [9]. CRM and LB assessed the quality of included papers using the Quality in Qualitative Evaluation framework and the NIH Quality Assessment Tool for Observational Cohort and Cross-sectional Studies [12, 13].

#### Analysis

We produced a narrative synthesis and review of the evidence. Findings reporting against the evaluation domains are presented descriptively. The heterogeneity of included papers did not allow for statistical meta-analysis.

## Results

**Fig. 1:**
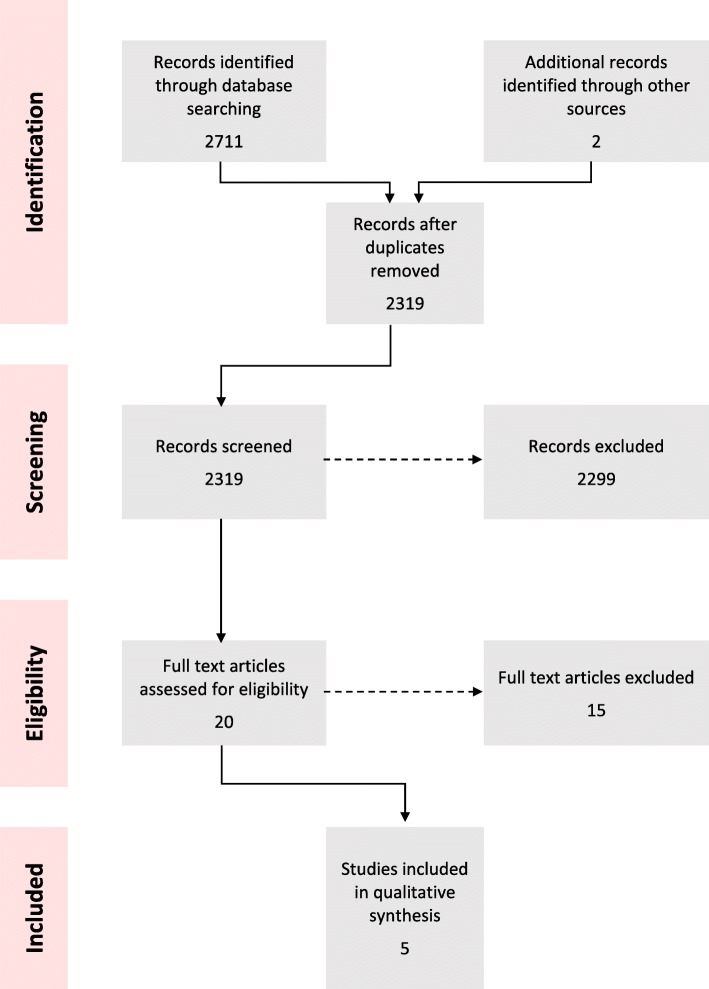
PRISMA Flow Chart

The database search yielded 2711 papers (Fig. 1). Five papers (describing five distinct studies) met the inclusion criteria and were included in the synthesis; these are summarised in Table 1 [14–18]. Papers were screened out following full-text assessment owing to: a lack of empirical data, the paper reported on an out of scope modality, and/or the context was a non-humanitarian setting. We also excluded three papers reporting on the Mobile Obstetric Maternal Health Workers (MOM) Project for internally displaced populations in eastern Burma. As this intervention involved mobile community health workers only, it was inconsistent with our definition of mobile clinics [19–21]. However, the authors agreed that the project’s evaluation methods were sound and may be adapted for mobile clinics providing maternal health services [19]. None of the grey literature sources met our inclusion criteria.

**Table 1:** Study characteristics

Study	Year	Country	Focus	Target population	Evaluation domain(s)	Outcome	Comparison	Quality
Al-Halaweh	2019	OPT	Non-communicable diseases	Adults with Type II diabetes in SW Bank Palestine.	Relevance/appropriateness, efficiency, and effectiveness	Glycaemic control	YES (Facility)	GOOD
Fils-Aime	2018	Haiti	Mental health	All care-seeking adults and children. Clinic was operated out of Kas, mainly serving Lahoye and Tierra Muscady in the Central Plateau.	Relevance/appropriateness, coverage, and effectiveness	Retention, care-seeking, depression symptom severity, stigma	NO	POOR
Kohli	2012	DRC	Multiple	Survivors of sexual and gender based violence in rural Walungu Territory, South Kivu.	Relevance/appropriateness, coverage, and effectiveness	Retention, access, patient satisfaction	NO	FAIR
Morikawa	2011	Afghanistan	Multiple	All care-seeking adults and children in three provinces in northern Afghanistan.	Coverage and effectiveness	Access	YES (Facility)	POOR
Phillips	2017	Haiti	Sexual and reproductive health	Pregnant women from 10 communes in the Central Plateau.	Efficiency and effectiveness	Quality of care, patient knowledge, patient perception of quality	YES (Facility)	GOOD

The five included studies reported on mobile clinic interventions focusing on non-communicable diseases [14], mental health [15], sexual and reproductive health [18], and multiple primary health services [16, 17] in Afghanistan [17], the Democratic Republic of the Congo (DRC) [16], Haiti [15, 18], and the Occupied Palestinian Territories (OPT) [14]. The study designs employed included: a quasi-experimental longitudinal study [14], two retrospective longitudinal studies [15, 17], a cross-sectional survey [9], and one self-described ‘case study’ which includes some elements of programme evaluation [16]. Three studies included a qualitative component (e.g. qualitative interviews, exit interviews) [15, 16, 18]. The quality of included studies was assessed as good [14, 18], fair [16], and poor [15, 17].

The five studies reported on four of the seven OECD-DAC evaluation criteria, namely relevance/appropriateness [14–16], coverage [15–17], efficiency [14, 18], and effectiveness [14–18]. All five studies sought to assess effectiveness to some extent. None of the papers reported substantively on connectedness, coherence, or impact.

### A comparative study of fixed and mobile clinics for delivering diabetes care model in the OPT

Al-Halaweh et al. assessed the *relevance/appropriateness, efficiency,* and *effectiveness* (defined as comparative improvements in various indicators of glycemic control) of the Diabetes Comprehensive Care Model (DCCM) delivered via a mobile diabetes care team in Hebron, OPT compared to ‘treatment as usual’ diabetes care delivered via a fixed facility in Bethlehem [14]. The Mobile Diabetes Clinic aimed to facilitate community-wide implementation of the DCCM model and, “…to create awareness of diabetes in the community as a way to improve control and prevent complications while building the clinical capacity of front-line staff and unifying management and care protocols”. ([14], p. 783–784).

The mobile diabetes clinic was staffed by a multi-disciplinary team of healthcare professionals and was equipped with screening and diagnostic equipment, as well as foot-care equipment. The mobile clinic team provided, “…a comprehensive diabetes assessment and care to patients and their families including counselling with diabetologist, nurses, and nutritionists to foster healthy lifestyle choices”. ([14], p., 783) The intervention is described as, “…a person-centered approach [which] requires community engagement and participation to foster diabetes awareness and prevention”, adapted for the local context in OPT. ([14], p., 783) The programme required patients to attend the mobile clinic every three months over the period of a year.

The study team comparatively evaluated diabetes care outcomes, as evidenced by measures of glycemic control, in two similar catchment populations in OPT. One hundred Type II diabetes patients were recruited through the fixed facility in Bethlehem, and 100 were recruited from the Mobile Diabetes Clinic in Hebron. The authors found that study participants attending the Mobile Diabetes Clinics demonstrated statistically significant improvements in diabetes control (as measured by HbA1c [*p* < .001], BMI [*p* = .030], serum creatinine [*p* = .028], and systolic blood pressure [*p* = .006]) compared to those receiving ‘treatment as usual’ via the fixed facility.

### Mobile clinics for providing mental health services in Haiti

Fils-Aime et al.(2018) assessed the *relevance/appropriateness*, *coverage*, and *effectiveness* of mental health mobile clinics in Haiti which aimed to, “overcome two major challenges to the provision of mental healthcare in resource-limited settings: the shortage of trained specialists; and the need to improve access to safe, effective, and culturally sound care in community settings”. ([15], p., 1) The mobile clinic intervention was developed in response to loss to follow-up in a mental health program that was implemented following the 2010 earthquake. A community-based intervention was deemed appropriate to address the region’s remoteness, high burden of mental health needs, and lack of access to alternative services. The clinic operated out of a small church every one to two months.

The study evaluated retention (as a proxy for “safe, effective, and culturally sound care”) which varied by diagnosis: bipolar disorder, movement disorder, and epilepsy/seizure had high follow-up rates (75, 73 and 65% respectively), but only a third of patients with depressive and anxiety disorders presented for follow-up. ([15], p., 1) The authors found no significant difference (*p* = 0.9) in follow-up by depression symptom severity. Of the patients who completed the quality-improvement questionnaire nearly half (47%) had never accessed care for their problem before attending the mobile clinic. Referrals were high with 20% of patients referred to a health centre, 10% for laboratory testing, 7% to a specialist, and 6% to more intensive mental health services [15].

### Mobile clinics for supporting survivors of gender-based violence (GBV) in the DRC

In 2004 the Congolese NGO Foundation RamaLevina (FORAL) started a mobile health programme for vulnerable women and men in order to address barriers to access identified by GBV survivors and their families in conflict-affected rural South Kivu, DRC [16]. Kohli et al. (2012) aimed to evaluate the *relevance/appropriateness*, *coverage*, and *effectiveness* of the mobile clinic and found that the programme, “…improved access to health care by survivors and their male partner, enhanced quality of health education, and facilitated regular monitoring, follow-up care and referrals”. ([16], p., 1) The programme was designed for survivors and other vulnerable women and girls and included socio-economic and reintegration services for survivors and their families as well as awareness education to reduce stigma in the community. The clinic services included health and hygiene promotion and sought to engage male partners in health care. Clinic activities included an interactive health education session followed by individual health care services for women. The clinic included laboratory testing (for HIV, syphilis, and HBV), and referrals. The clinic rotated through six villages.

Approximately 72% of patients returned for their first follow-up visit, with attendance dropping for second (7%) and third (3%) follow-up visits. Nearly half of the women (45%) attending the mobile clinic between July 2010 and June 2011 reported not receiving health care services after their last sexual assault. Local community health workers reported that the clinic gave them confidence to provide village members with accurate information on STI/HIV prevention and other topics.

Efforts by mobile clinic staff to protect the identity of survivors of GBV, build relationships with patients, and provide targeted health education, “…contributed to patient appreciation of the compassionate, non-judgmental and high-quality care received at the FORAL mobile clinic”. ([16], p., 6) Younger women reported not accessing mobile clinic services out of fear of being seen by older women who could be their future mother-in-law and who may limit their opportunities for marriage due to the perception that the women were sexually active. This case study illustrated: “ [1] that more frequent visits may improve provider-patient communication and relationships and allow for targeted health education, care and treatment to survivors of GBV and male partners [2]; the importance of local partnerships to avoid redundancy and increasing opportunities for leveraging and sustaining efforts [3]; the value of a monitoring and evaluation system to improve services; and [4] the need for locally relevant and sustainable psychosocial services for survivors and other members of the community, including male partners”. ([16], p., 8)

### A comparative study of fixed and mobile clinics for providing health services in northern Afghanistan

Morikawa et al. (2011) evaluated the *coverage* and *effectiveness* of mobile clinics in northern Afghanistan (defined as a comparison of seasonal variations in attendance between fixed clinics and mobile clinics) [17]. The mobile clinics aimed to reduce "markedly high" maternal and child mortality by providing free services for internally displaced persons and patients in remote rural areas with insufficient access to primary care; particularly, malnourished children, pregnant women, and newborn infants. Four mobile teams (each staffed by one doctor, one midwife, and one nurse) provided medical services in remote villages as well as transportation of seriously ill patients to referral facilities.

The seasonality of fixed clinic visits was compared to visits to the mobile clinics. The study demonstrated a considerable drop in visits to the fixed clinics during winter months; however, no such variation was observed for the mobile clinics suggesting that ease of access to the mobile clinic during the winter months may have enabled uptake of services. The study team concluded that, “[c]onsidering the tenuous access during the winter months in northern Afghanistan, access of care would be better addressed by mobile teams than regular clinics”. ([17], p., 58)

### A comparative study of fixed and mobile clinics for providing antenatal care in Haiti

Phillips et al. (2017) compared the *efficiency* and *effectiveness* of antenatal care (ANC) between fixed and mobile clinics in Haiti as measured by eight components of care, as well as women’s knowledge and perception of quality of care [18]. The Maternal and Child Health and Nutrition Program (MCHNP) provided free ANC and post-natal care, with the intention of increasing ANC coverage, via 130 mobile clinics in locations in the Central Plateau of Haiti with limited access to health services. Unlike many mobile clinics, the MCHNP clinics were not implemented as a short-term solution to poor health service coverage, but rather were implemented on a large scale over a protracted period of time supported by successive grants. The clinics were held monthly and were staffed with 40 health professionals including auxiliary nurses and nurse-midwives. Services also included behaviour change communication, growth monitoring, vaccination for children under five, and food distribution.

The assessment of quality of care was based on the percentage of completeness of services provided under each of eight care components (i.e. intake, physical exam, laboratory exam, distribution of supplies, iron-folic acid and tetanus toxoid vaccine, educational messages and counseling, health provider communication and interpersonal delivery, infection prevention and control [IPC], and documentation). The authors conclude that, overall, the quality of ANC care was weak in both delivery models owing to the low percentage of possible services delivered in six of the eight care components. However, there were some significant differences between delivery models: lab exams, and IPC components were delivered more frequently in fixed clinics. Compared to the fixed facilities, there was a smaller proportion of instances of hand sanitisation and proper disposal of medical waste in the mobile clinics. As such, the study team concluded that, “…mobile clinics can provide similar quality of ANC as fixed clinics in the majority of care components studied” but that “…the lack of laboratory exams offered in the mobile clinics is a potential structural weakness of the model, as carried out in this context”. ([18], p., 8)

The study also explored the effectiveness of health messaging delivered as part of their clinic visits and determined that women who attended mobile clinics were more likely to recall the correct recommended duration of breastfeeding and danger signs. A perception of having received high-quality of care was similar for both fixed and mobile clinics. Ultimately, the study concluded that “…the quality of ANC delivered through mobile clinics suffers from similar problems as fixed clinics in central Haiti”. ([18], p., 9)

### Summary of recommendations

Four of the five studies included recommendations for improving or evaluating the mobile clinic modality. Fils-Aime et al. suggest that mobile mental healthcare services should complement clinic-based services in resource limited settings owing to barriers to the provision of services in fixed clinics [15]. The authors further suggest that technology-based innovations could help to improve follow-up, communication and coordination of care, and medical record keeping [15]. Kohli et al. identified three areas for development – though these are not necessarily specific to mobile clinics - including, “provision of health services to young, unmarried women in a way that reduces possibility of future stigma, engaging male partners in health education and clinical care, and strengthening linkages for referral of survivors and their partners to psychosocial support and mental health services” [16]. Finally, Phillips et al. highlight that the absence of laboratory testing is a potential structural weakness of mobile clinics. However, they further note that this limitation does not negate the potential benefits but suggests the need for, “…an integrated system of ANC with strong coordination of care between mobile and fixed clinics to optimize the continuum of care”. ([18], p., 8). Recommendations regarding evaluation include ensuring that beneficiaries of mobile clinic services and those in the comparison group receive the same clinical contact in order to test the effectiveness of the modality, rather than the clinical service [14].

## Discussion

This review identified a limited evidence base on the use of mobile clinics in humanitarian responses, with available studies covering divergent settings, service packages, and evaluation domains. Moreover, evaluation methods were mostly ill-suited to isolate the effect of the mobile clinic modality on outcomes. There is little to no published evidence on connectedness (i.e. how the intervention is woven into the setting and long-term programmes), coherence with humanitarian policy and the wider response, or impact. Nevertheless, available studies suggest that mobile clinics may be relevant/appropriate, efficient, effective, and increase service coverage. One study concluded that mobile clinics did not demonstrate improved quality of care when compared to fixed facilities; another demonstrated poor follow-up [15, 18].

### Improving the relevance/appropriateness of mobile clinics

Several considerations underpin the design of an appropriate package of primary health services for mobile clinics. First, the frequency with which the same communities can be visited will determine whether the mobile clinic should offer care and/or referral for acute illnesses (e.g. acute respiratory infections, acute diarrhoea, malaria, neonatal and maternal emergencies). Modelling suggests that unless mobile clinics are able to provide daily curative services, a fixed community health post or community case management approach will be more effective for reducing mortality due to childhood pneumonia [22]. Indeed, there is a risk that infrequent or unpredictable visits by mobile clinics may discourage communities from seeking early care at the nearest fixed facility, and thus worsen acute illness outcomes.

Second, mobile clinics are comparatively better suited to offer preventive services (e.g. vaccination, antenatal care) or outpatient-level case management of chronic conditions (e.g. mental health problems, high-burden non-communicable diseases). The most appropriate configuration of services for the local conditions should be planned carefully, and must be based on the local burden of disease and crisis-emergent risk factors. It is unlikely in most crisis settings that mobile teams will be able to deliver a comprehensive package of preventive and curative care; as such, clear prioritisation criteria could be applied to select the most impactful subset that is deliverable given local constraints.

### Additional relevance/appropriateness considerations

The descriptions of the services in each of the five included studies suggest the absence of several design components relating to relevance/appropriateness which may be well-suited to a mobile clinic modality. In the absence of robust evidence to inform the design of mobile clinics for implementation in humanitarian emergencies we suggest the integration of the following components:

#### Water supply, sanitation, and hygiene promotion (WASH) services

In remote communities, mobile clinics are likely to attract large numbers of people, which in turn creates a considerable risk of nosocomial infections. To avoid this harm, all mobile clinic designs could include IPC and facility WASH services for both staff and patients (patient spacing, gloves and other barriers, clean drinking water, temporary pit latrines, soap, portable hand washing stations, and safe disposal of highly pathogenic excreta). Mobile clinics have the potential to provide a useful platform for delivering WASH-related hygiene information, education, and communication (IEC) materials, household WASH supplies (e.g. chlorination kits), and routine maintenance of local WASH infrastructure (e.g. water pumps, latrines) if mobile clinic staff are joined by WASH technicians.

#### Nutrition services

Humanitarian emergencies increase the burden of acute malnutrition through food insecurity, increased disease burden, poor WASH, and compromised care practices. Without adequate support, infant and young child feeding (IYCF) practices can worsen, leaving the youngest children particularly vulnerable to malnutrition, disease, and death. Whilst interventions to identify and treat acute malnutrition are effective, it is often difficult for caretakers with young children to travel long distances to access services that offer nutritional care [1, 23]. Mobile clinic services may therefore promote equity and greater access to essential nutrition services. We encourage humanitarian actors to implement anthropometric screening of children under five years, and pregnant and breastfeeding women in all outpatient settings in which there is high nutrition vulnerability, with referral to community management of acute malnutrition (CMAM) services and infant and young child feeding support where indicated. Moreover, treatment of children who have severe acute malnutrition (SAM) - which is provided on an outpatient basis for children presenting without medical complications and which requires weekly follow-up contact (where the context allows) with children and their caregivers - may be deliverable via mobile clinics if mobile teams can access the same communities with predictable recurrence [24]. Exhaustive screening for acute malnutrition could also be carried out at the population level by trained home visitors accompanying the mobile team. Finally, infant and young child feeding assessments and counselling for caretakers of children under two years may be carried out during mobile clinic visits, thereby increasing uptake of optimal feeding and care practices, a key protective factor for early child survival [23].

#### Epidemic surveillance

An effective disease surveillance system is essential to detecting disease outbreaks quickly before they spread and become difficult to control. Early Warning and Response systems (EWARS) are designed to improve disease outbreak detection in emergency settings, such as in countries in conflict or following a natural disaster. Mobile clinics could be added to the network of facilities reporting to any existing EWARS, and may have a particular advantage over fixed facilities in detecting initial outbreak clusters, thereby enabling containment and/or rapid response.

#### Quality and safety of patient care

Whilst mobile clinics can contribute to rapidly expanding the reach of health services, there is a risk that quality and safety of these services may be neglected in the process. Mobile clinics may face more challenges than fixed services in implementing quality assurance processes, particularly if they are moving frequently between sites and do not have a stable catchment population.

Forty percent of patients are estimated to be harmed to some extent when accessing primary and community care [25]. This may include misdiagnosis, inappropriate prescriptions, injury, or death. All health services therefore require a systematic approach to maintaining and improving the quality and safety of patient care, and mobile clinics should not be an exception. ([26], p., 14) This systematic process of assuring quality and safety is referred to as clinical governance and core components of this usually include: a patient charter, a health information and clinical incident management system, auditing processes, the use of evidence-based case management protocols, clinical supervision and training processes, and staff and patient feedback services (the latter accountability mechanisms, including client exit interviews, are logistically straightforward and can also be used to evaluate the effectiveness of mobile clinics). All of these must sit within a culture of continuous improvement of care.

### Recommendations for further evidence generation

We encourage humanitarian actors who have carried out evaluations of their mobile clinic interventions to publish their findings. Findings would ideally follow an established framework (e.g. the adapted DAC criteria or the Core Humanitarian Standard) and report on multiple domains, particularly those that are commonly overlooked or which can be difficult to evaluate (i.e. connectedness, coherence, and impact) [9, 27].

There is a need to develop standard indicators - ideally co-produced with communities who use mobile health services - for evaluating the use of mobile clinics in emergencies. Indicators may be monitored throughout the duration of programming to enable course correction and effective transition of services to fixed facilities. However, we encourage the use of longitudinal studies as cross-sectional studies often fail to indicate whether programmes are contributing to medium or long-term outcomes for crisis-affected people. [28] Finally, we encourage routine collection, meaningful interpretation, and dissemination of qualitative data based on clearly defined evaluation domains. We encourage operational partners to involve affected communities in the design and conduct of evaluation studies to ensure that studies are meaningful and adequately capture community experiences of mobile health clinics.

### Limitations of the review

Our search did not cover non-humanitarian settings and has, thus, not included evidence that may be partly transferable to the humanitarian context. Similarly, we have not identified evaluations other than those published in scientific outlets. The small number of eligible reports may reflect a bias towards positive results. Positive outcomes may also be influenced by authors’ personal connection to the intervention being evaluated.

## Conclusion

There are few published studies evaluating the use of mobile clinics in humanitarian emergencies despite the fact that they are a common modality for delivering health services in such settings. This review identified five studies evidencing the relevance/appropriateness, coverage, efficiency, and effectiveness of mobile clinics in humanitarian settings; no studies reported on connectedness, coherence, or impact. Only two studies were determined to be of good quality. Four studies made recommendations including: i) ensure that mobile clinics are designed to complement clinic-based services; ii) improve technological tools to support patient follow-up, record-keeping, communication, and coordination; iii) avoid labelling services in a way that might stigmatise attendees; iv) strengthen referral to psychosocial and mental health services; v) partner with local providers to leverage resources; and vi) ensure strong coordination to optimise the continuum of care. Recommendations regarding the evaluation of mobile clinics include carrying out comparative studies of various modalities (including fixed facilities and community health workers) in order to isolate the effect of the mobile clinic modality.

In the absence of a sound evidence base informing the use of mobile clinics in humanitarian crises, we encourage improving their relevance/appropriateness through the integration of: i) WASH services, ii) nutrition services, iii) epidemic surveillance, and iv) systems to ensure the of quality and safety of patient care. Rigorous evaluations are needed, and we encourage operational partners to publish evaluations in order to inform the evidence base for best practice.

Finally, we draw attention to findings which suggest that mobile clinics may not perform better than fixed facilities in some settings [16]. Mobile clinics may seem like an attractive default modality of care delivery due to various factors (donor support, rapidity of implementation, ability by the humanitarian actor to ‘control’ service provision, media impact, and fundraising potential). However, it is essential that an objective options appraisal be conducted before settling on this approach: in particular, in many settings more evidence-based, cost-effective, and sustainable options may exist that may also better support local health systems. For example, revitalising disrupted or dormant networks of community health workers by providing them with supplies and supervision to carry out community case management of key acute illnesses; establishing simple fixed health posts with mobile teams instead serving a resupply, supervisory, or referral/ambulance function; or directly addressing barriers to accessing existing fixed facilities (e.g. through cash transfers for community transport) [29, 30].

## Data Availability

Not applicable.

## Abbreviations

ANC: Ante-natal care
CMAM: Community management of acute malnutrition
DCCM: Diabetes Care Collaborative Model
DRC: Democratic Republic of the Congo
EWARS: Early warning, alert and response system
FORAL: Foundation Rama Levina
IEC: Information, education, and communication
IYCF: Infant and young child feeding
MeSH: Medical subject heading
OECD-DAC: Organisation for Economic Co-operation and Development - Development Assistance Committee
OPT: Occupied Palestinian Territory
SAM: Severe acute malnutrition
WASH: Water supply, sanitation, and hygiene promotion
